# Daily activity, mood, and quality of life in colorectal cancer patients with chemotherapy‐induced peripheral neuropathy: A mediation effect analysis

**DOI:** 10.1002/cam4.1976

**Published:** 2019-02-11

**Authors:** Ling‐Chun Lu, Shiow‐Luan Tsay, Sou‐Yi Chang, Chung‐Ming Chen, Chieh‐Yu Liu

**Affiliations:** ^1^ School of Nursing National Taipei University of Nursing and Health Sciences, Nurse Practitioner, Koo Foundation, Sun Yat‐Sen Cancer Center Taipei City Taiwan (R.O.C.); ^2^ College of Nursing and Health Sciences Da‐Yeh University Changhua County Taiwan (R.O.C.); ^3^ Department of Hematology and Oncology Taipei Tzu Chi Hospital New Taipei City Taiwan (R.O.C.); ^4^ Department of Colorectal Surgery Hualien Tzu Chi Hospital Hualien County Taiwan (R.O.C.); ^5^ Biostatistical Consultant Lab, Department of Speech Language Pathology and Audiology National Taipei University of Nursing and Health Sciences Taipei City Taiwan (R.O.C.)

**Keywords:** chemotherapy‐induced peripheral neuropathy, daily activity, mediation effect analysis, mood, quality of life

## Abstract

Chemotherapy‐induced peripheral neuropathy (CIPN) with restriction of daily activity (RDA) was common consequence of oxaliplatin‐based chemotherapy in colorectal cancer patients. CIPN with RDA and negative mood may impact the quality of life (QoL). However, the relationships among RDA, mood, and QoL remain unclear. This was a cross‐sectional relative study in which four instruments were used: the Neuropathic Pain Symptom Inventory was used to measure the severity of CIPN; the Screening of Activity Limitation and Safety Awareness scale was used to evaluate RDA; the Profile of Mood States Short Form was used to assess negative mood; and the Functional Assessment Cancer Center Therapy‐Colorectal scale version 4 was used to evaluate QoL. Relationships among the variables were analyzed by bivariate correlation, hierarchical multiple linear regression, and Baron and Kenny's mediation testing. One hundred three colorectal adenocarcinoma patients with CIPN after receiving oxaliplatin‐based chemotherapy were enrolled. Patients had mild‐to‐moderate CIPN and mild RDA. Significant correlations were found between CIPN and mood (*r* = 0.425, *P *< 0.001), between RDA and mood (*r* = 0.343, *P *< 0.001), and between RDA and QoL (*r* = 0.285, *P *< 0.01). RDA and mood may impact QoL. Under mediation effect analysis, mood mediated 38.48% of the effect of RDA on QoL (*P* < 0.001). Negative mood is the major factor impacting QoL in colorectal cancer patients with CIPN. Although the management of CIPN and RDA can prevent irreversible functional problems, enhancing the adaption of mood disturbance can strongly promote their QoL.

## 1 INTRODUCTION

Colorectal cancer is the third most common cancer in terms of incidence and the fourth most common cancer in terms of mortality wordwide.^1^ Oxaliplatin‐based chemotherapy is beneficial in the treatment of colorectal cancer in adjuvant, advanced and metastatic settings.^2^ After patients receive the therapy, approximately 72%‐95.2% of them consequently experience acute or chronic peripheral neuropathy.^3^, ^4^ These acute peripheral neuropathic symptoms may develop after the first course of treatment but may be relived spontaneously between courses.^4^, ^5^ When the total accumulative dose of oxaliplatin is approximately 540‐850 mg/m^2^, the symptoms may last for months or years.^3^, ^5^, ^6^^.^ Acute peripheral neuropathic symptoms in the limbs include paresthesia, dysesthesia, neuropathic pain, painful muscle spasm, and evoked hypersensitivity as touching cold objects. Chronic peripheral neuropathic symptoms in the limbs comprise persistent paresthesia, dysesthesia, altered proprioception, sensory ataxia, progressive sensory loss, and decreased vibratory and temperature sensation.^3^, ^4^, ^5^, ^6^, ^7^ When patients had chemotherapy‐induced peripheral neuropathy (CIPN) in limbs, the condition often concurred with restriction of daily activity (RDA).^5^, ^8^, ^9^

When CIPN occurs in the upper limbs, patients may have interference with dressing, household chores, hobbies, and work.^9^ As they wear clothing, they may have problems with buttoning, using zippers, fastening brassieres, tying neckties, and putting on earrings or jewelry.^5^, ^10^ They may have difficulty in opening jars, removing cold objects from the refrigerator, threading a needle, using remote controls or controllers for video games or picking up small objects from the ground.^10^, ^11^ When CIPN develops in the feet, the patient's walking, climbing stairs, hiking, running, biking, exercise, or standing for a long time may be interfered.^10^ The evoked hypersensitivity as touching cold objects causes patients to not go barefoot, and the paresthesia, dysesthesia, neuropathic pain, painful muscle spasm, and leg weakness lead to imbalance.^9^, ^10^, ^11^ Thus, the risk of falls increases in colorectal cancer patients with CIPN.^12^

The impact of CIPN on mood or quality of life (QoL) has been clearly demonstrated in several studies.^11^, ^13^, ^14^ Patients with CIPN often also show RDA; however, the relationship among RDA, mood, and QoL remains unclear in colorectal cancer patients with CIPN.^8^, ^10^, ^13^, ^14^, ^15^, ^16^ When patients with CIPN also show functional limitations, such as slipping, falling or abnormal walking, they often express annoyance, irritability, weariness, frustration, anger, and depression.^10^ Patients with CIPN and functional limitation show poorer emotional status and QoL.^8^, ^13^, ^14^ When patients with CIPN develop social limitation, such as withdrawal from hobbies, leisure, work or family roles, they experience emotional distress.^10^, ^11^ Cancer patients with anxiety, depression, and hopelessness may show effects on QoL.^15^ In addition, cancer patients with depression and anxiety showed significantly poorer QoL.^16^, ^17^

The relationships among RDA, mood, and QoL in colorectal cancer patients with CIPN seem to be complex. The aim of this study was to investigate the relationships among RDA, mood, and QoL in colorectal cancer patients with CIPN.

## 2 METHODS

### 2.1 Participants and procedure

This is a cross‐sectional relative study. Patients who were hospitalized at two cancer centers in Taiwan in 2011 were recruited. The inclusion criteria were as follows: (a) diagnosis of adenocarcinoma by surgical pathology and the cancer located in the colon or rectum; (b) received oxaliplatin‐based chemotherapy; (c) new peripheral neuropathic symptoms in the extremities after receiving oxaliplatin‐based chemotherapy; and (d) ability to understand and answer the questionnaires as well as ability to obey requirements to assess RDA. In the study, the symptoms of CIPN in the limbs included paresthesia, dysesthesia, neuropathic pain, painful muscle spasm or weakness, and hypersensitivity to cold objects. This study was approved by the Institutional Review Boards of Koo Foundation, Sun Yat‐Sen Cancer Center and Taipei Tzu Chi Hospital (Approval No: 00‐IRB‐005‐M). Signed informed consent was obtained from all participants before data collection. Four of the one hundred seven recruited patients refused the invitation because of poor concentration and fatigue related to chemotherapy.

### 2.2 Instruments

Four instruments were included in the study: (a) the Neuropathic Pain Symptoms Inventory (NPSI) was used to measure the severity of various CIPN symptoms, (b) the Screening of Activity Limitation and Safety Awareness (SALSA) scale was used to evaluate RDA, (c) the Profile of Mood States Short Form (POMS‐SF) was used to assess negative mood, and (d) the Functional Assessment Cancer Center Therapy‐Colorectal scale (FACT‐C) version 4 was used to evaluate QoL.

#### 2.2.1 Neuropathic Pain Symptoms Inventory

NPSI was established by Bouhassira and colleagues in 2004.^18^ The inventory consists of 12 items and four subscales. The four subscales are spontaneous pain, paroxysmal pain, evoked pain, and dysesthesia/paresthesia. Ten of the items are quantified on a 0‐10 numerical scale to evaluate the severity of diverse neuropathic pain symptoms during the past 24 hours. The other two items have 5‐point Likert‐type questions to evaluate the duration of spontaneous pain and frequency of paroxysmal pain, respectively. Higher scores on the total scale indicate more severity of neuropathy. Its reliability was high (Cronbach's *α* coefficient of the total scale was 0.90), and the construct validity by factor analysis was significant (*P* < 0.001).^18^ The scale also demonstrated adequate reliability and content validity for evaluating patients with peripheral neuropathic pain in the United States, Brazil, Japan, China, Finland, and Spain.^19^ The reliability of the Chinese version of the NPSI for evaluating oxaliplatin‐induced peripheral neuropathy was high (Cronbach's *α* coefficient of the total scale was 0.90), the construct validity by confirmatory factor analysis with goodness‐of‐fit indices, included normed chi‐square (*χ* ^2^/*df*), root mean square error of approximation (RMSEA), root mean square error with respect to the mean (RMSEM), goodness‐of‐fit index (GFI), comparative fit index (CFI), and incremental fit index (IFI), was significant (*χ^2^/df* = 1.78; RMSEA = 0.08; RMSEM = 0.085, 90% confidence interval (CI), 0.041‐0.12; GFI = 0.92; CFI = 0.96; IFI = 0.96), and the convergent validity was significant with respect to mood (*P* < 0.05).^7^

#### 2.2.2 Screening of the Activity Limitation and Safety Awareness Scale

The SALSA scale was established by the SALSA Collaborative Study Group in 2007.^20^ The scale consists of 20 items and four subscales. The four subscales are mobility of foot, self‐care, work of hand, and dexterity of hand. Twenty of the items have 5‐point (0‐4) Likert‐type questions to evaluate the limitation of daily activity in limbs. Higher scores on the total scale represent more severe RDA. The reliability was significant for evaluating diabetic peripheral neuropathy (Kappa values ranged from 0.45 to 0.8).^20^, ^21^ The reliability of the Chinese version of the SALSA scale for evaluating oxaliplatin‐induced peripheral neuropathy was acceptable (the Cronbach's *α* coefficient of the total scale was 0.70), the content validity was good (item‐level content validity indexes were 0.8‐1.0; the average scale‐level CVI was 0.89), and the criterion‐related validity was significant with respect to mood (*P* < 0.01) and QoL (*P* < 0.05).^22^

#### 2.2.3 Profile of Mood States Short Form

POMS‐SF was developed from the 65‐item Profile of Mood States scale by Shacham in 1983.^23^ The scale is a self‐administered questionnaire containing 30 items. The 30 items are a set of 5‐point (0‐4) adjective rating scales with a six‐bipolar‐factor structure. The six bipolar factors are tension‐anxiety, depression‐dejection, anger‐hostility, vigor‐activity, fatigue‐inertia, and confusion‐bewilderment. Higher scores on the total scale indicate a more negative mood. The reliability of the Chinese version of POMS‐SF was very high (Cronbach's *α* coefficients were 0.98‐0.99), and the validity by factor analysis was significant (*P* < 0.05).^24^, ^25^

#### 2.2.4 Functional Assessment Cancer Center Therapy‐Colorectal scale version 4

The FACT‐C version 4 was evaluated by Yoo and colleagues in 2005, which is a self‐administered questionnaire.^26^ The instrument consists of 36 items and five domains. The five domains are physical well‐being, social well‐being, emotional well‐being, functional well‐being, and the colorectal cancer subscale. The 36 items have 5‐point (0‐4) Likert‐type questions. Higher scores on the total scale indicate better QoL. The reliability (Cronbach's *α* coefficients were approximately 0.81‐1.0) and concurrent validity of the Chinese version of the FACT‐C version 4 significantly correlated with those of EORTC OLQ‐C30/CR38 and SF‐12 v2 (*r* ≧ 0.4).^27^

### 2.3 Data analysis

The data were analyzed using SPSS software (version 19.0; SPSS Inc, Chicago, IL). A descriptive basis (percentage distributions, means and standard deviations) was used to calculate the patients’ characteristics and instrument scores. The bivariate correlations among CIPN, RDA, mood, and QoL were analyzed by Pearson's correlation coefficients. Hierarchical multiple linear regression analysis was used to measure the effects of CIPN, RDA and mood on QoL. When CIPN was set on covariates, medication effect analysis with multiple linear regressions was based on Baron and Kenney's method.^28^, ^29^

## 3 RESULTS

### 3.1 Patients’ characteristics

The patients’ characteristics are summarized in Table 1. Most of the patients were aged 61‐70 years (34%), were male (55.3%), were married (79%), had a high school education level (45.6%), left work (63.1%), had stage III disease (60.2%), had hypertension (25.2%), had diabetes mellitus (15%), had spinal disease (5.8%), and had an accumulation dose of chemotherapy above 541 mg/m^2^ (56.3%).

**Table 1:** Patient characteristics (N ＝ 103)

Characteristics	n	%
Age in years		
0‐30	2	1.9
31‐40	5	4.9
41‐50	25	24.3
51‐60	26	25.2
61‐70	35	34.0
71 or above	10	9.7
Gender		
Male	57	55.3
Female	46	44.7
Marital status		
Married	79	76.7
Single	24	23.3
Education		
Primary school	31	30.1
High school	47	45.6
University	25	24.3
Occupation		
None	65	63.1
Yes	38	36.9
Stage		
II	6	5.8
III	62	60.2
IV	35	34.0
Chronic illness		
Hypertension	26	25.2
Diabetes mellitus	15	14.6
Spinal disease	6	5.8
Accumulation dose (mg/m^2^)		
0‐540	45	43.7
541 or above	58	56.3

### 3.2 Scores of CIPN, RDA, mood, and QoL

The mean NSPI score was 13.66 (SD, 12.05; range, 1‐68). Scores of the subscales on spontaneous neuropathic pain, paroxysmal neuropathic pain, evoked neuropathic pain, and dysesthesia/paresthesia were 1.95 (SD, 3.663), 2.17 (SD, 3.359), 4.90 (SD, 4.756), and 4.63 (43.25). Evoked neuropathic pain, especially that evoked by cold stimulation, and paresthesia (numbness/tingling) were the two major discomforts.

The mean SALSA scale score was 19.50 (SD, 4.228; range, 11‐33). Scores of the subscales on mobility of foot, self‐care, work of hand, and dexterity of hand were 3.44 (SD, 1.667), 3.29 (SD, 0.946), 6.83 (SD, 2.540), and 4.79 (1.348). The ranking of the SALSA subscales from high to low scores was work of hand, dexterity of hand, mobility of foot, and self‐care.

The mean POMS‐SF score was 28.92 (SD, 13.790; range 5‐67). Of the 6 subscales of the POMS‐SF, the vigor‐activity subscale had the highest mean score. Scores of these subscales on tension‐anxiety, depression‐dejection, anger‐hostility, vigor‐activity, fatigue‐inertia, and confusion‐bewilderment were 3.49 (SD, 3.534), 3.21 (SD, 2.996), 2.82 (SD, 2.882), 10.88 (SD, 4.453), 4.14 (SD, 3.106), and 4.39 (2.474).

The mean score on the FACT‐C version 4 was 100.11 (SD, 15.877; range 60‐134). The scores of subscales on physical well‐being, social well‐bing, emotional well‐being, functional well‐being, and colorectal cancer subscale were 22.65 (SD, 4.08), 21.00 (SD, 40 548), 19.43 (SD, 3.706), 17.37 (SD, 5.792), and 19.66 (4.281). Among the 5 dimensions of the FACT‐C version 4, the domain of physical well‐being had the highest mean score.

### 3.3 Relationship among RDA, mood and QoL in colorectal patients with CIPN

The bivariate correlation between CIPN, RDA, mood, and QoL is listed in Table 2. There was a significantly positive relationship between paroxysmal neuropathic pain and self‐care (*r* = 0.249, *P *< 0.05) as well as between dysesthesia/paresthesia and dexterity of hand (*r* = 0.95, *P *< 0.05). CIPN and negative mood showed a moderate significant positive correlation (*r* = 0.425, *P *< 0.001), but only a mild, significant negative correlation was observed between physical well‐being and QoL (*r *= −0.233, *P *< 0.05). The RDA showed a intermediate positive correlation with mood (*r* = 0.343, *P *< 0.001) and a mild negative correlation with QoL (*r* = 0.285, *P *< 0.01).

**Table 2:** Correlation among CIPN, RDA, mood, and QoL

	NPSI					SALSA scale				
	TS	SNP	PNP	ENP	DP	TS	MF	SC	WH	DH
SALSA scale										
TS	0.095	0.049	0.013	0.093	0.111					
MF	0.022	0.018	−0.028	−0.068	0.141					
SC	0.130	0.013	0.249*	0.054	0.098					
WH	−0.008	0.015	−0.146	0.125	−0.056					
DH	0.151	0.085	0.106	0.064	0.195*					
Mood										
TS	0.479***	0.425***	0.413***	0.311**	0.307***	0.343***	0.174	0.154	0.253*	0.206*
TA	0.372***	0.318**	0.315**	0.260***	0.234*	0.285**	0.025	0.268**	0.234*	0.160
DD	0.315**	0.340*	0.284**	0.228*	0.117	0.255**	0.109	0.116	0.222*	0.104
AH	0.334**	0.319**	0.360***	0.128	0.237*	0.135	0.209*	0.139	−0.018	0.035
VA	0.315**	0.250*	0.167	0.247*	0.261**	0.334**	0.233*	0.001	0.269**	0.239
FI	0.397***	0.384***	0.365*	0.203*	0.269**	0.261**	0.136	0.100	0.223*	0.223*
CB	0.303**	0.197*	0.333***	0.240*	0.152	0.112	−0.030	0.048	0.065	0.065
FACT‐C version 4										
TS	−0.148	−0.269*	−0.099	−0.058	−0.043	−0.285**	−0.129	−0.176	−0.257**	−0.077
PWB	−0.233*	−0.229	−0.132	−0.088	−0.254*	0.191	−0.072	0.017	−0.203*	−0.096
SWB	−0.240	−0.126	0.018	−0.014	0.042	−0.165	−0.134	−0.114	0.109	−0.023
EWB	−0.112	−0.169	−0.099	−0.067	−0.018	−0.346***	−0.179	−0.299**	−0.237*	−0.186
FWB	−0.030	−0.151	0.018	0.010	0.019	−0.155	0.035	−0.008	−0.255**	−0.003
CCS	−0.126	−0.287	−0.144	−0.036	0.044	−0.099	−0.055	−0.138	−0.105	0.084

AH, anger‐hostility; CB, confusion‐bewilderment; CCS, colorectal cancer subscale; CIPN, chemotherapy‐induced peripheral neuropathy; DD, depression‐dejection; DH, dexterity of hand; DP, dysesthesia/paresthesia; ENP, evoked neuropathic pain; EWB, emotional well‐being; FACT‐C, Functional Assessment Cancer Center Therapy‐Colorectal scale; FI, fatigue‐inertia; MF, mobility of foot; NPSI, Neuropathic Pain Symptoms Inventory; PNP, paroxysmal neuropathic pain; PWB, physical well‐being; QoL, quality of life; RDA, restriction of daily activity; SALSA, Screening of Activity Limitation and Safety Awareness; SC, self‐care; SNP, spontaneous neuropathic pain; SWB, social well‐being; TA, tension‐anxiety; TS, total score; VA, vigor‐activity; WH, work of hand.

* *P* < 0.05

** *P* < 0.01

*** *P* < 0.001

Hierarchical multiple regression analysis was used to calculate the effect of CIPN, RDA and mood on QoL (Table 3). Model I showed a 2.2% variance of CIPN with no significant effect on QoL (*F* ＝ 2.2743, *P* > 0.05). Model II showed a 9.6% variance of CIPN, and RDA showed a mild significant effect on QoL (*F* ＝ 5.316, *P* < 0.05). Model III showed a 38.5% variance of CIPN, and RDA and mood showed a strong, significant effect on QoL (*F* ＝ 20.645, *P* < 0.001).

**Table 3:** Relationships between CIPN, RAD, mood, and QoL

Model	Variables	*R^2^*	95% CI		*F*	*β*	*SE*	*t*
			Lower	Upper				
Hierarchical multiple regression analysis of CIPN, RAD, Mood, and QoL								
Model I		0.022	98.102	107.468	2.274	102.785	2.361	
	CIPN					−0.196	0.130	−1.508
Model II		0.096*	108.049	136.611	5.316*	122.330	2.361	
	CIPN					−0.162	0.126	−1.281
	RDA					−1.027	0.359	−2.863**
Model III		0.385***	112.542	136.257	20.645***	124.400	5.976	
	CIPN					0.119	0.119	1.895
	RDA					−0.292	0.316	−0.924
	Mood					−0.750	0.110	−6.817***
Mediation effects between RDA and Mood on QoL								
Mood mediates the effect of RDA on QoL		0.3848	−1.3375	−0.2254	20.6449***			
RDA (predictor) → Mood (mediator)						0.9799	0.2704	3.6236**
Mood(mediator) → QoL (outcome)						−0.7496	0.1100	−6.8168*
RDA (predictor) → QoL (outcome)						−1.0267	0.3586	−2.8628**
RDA (predictor) → QoL(outcome)|Mood (mediator)						−0.2922	0.3163	−0.9239
Control covariance (CIPN)						0.2256	0.1191	1.8950
RDA mediates the effect of Mood on QoL		0.1241	0.0353	−0.0981	7.0826			
Mood (predictor) → RDA (mediator)						0.1184	0.0327	3.6236***
RDA (mediator) → QoL (outcome)						−0.2922	0.3163	−0.9239
Mood (predictor) → QoL (outcome)						−0.7496	0.1100	−6.8168*
Mood (predictor) → QoL(outcome)|RDA (mediator)						16.5009	0.9206	17.9234***
Control covariance (CIPN)						−0.0316	0.0375	0.4018

CIPN, chemotherapy‐induced peripheral neuropathy; QoL, quality of life; RDA, restriction of daily activity.

* *P* < 0.05

** *P* < 0.01

*** *P* < 0.001

**Figure 1:**
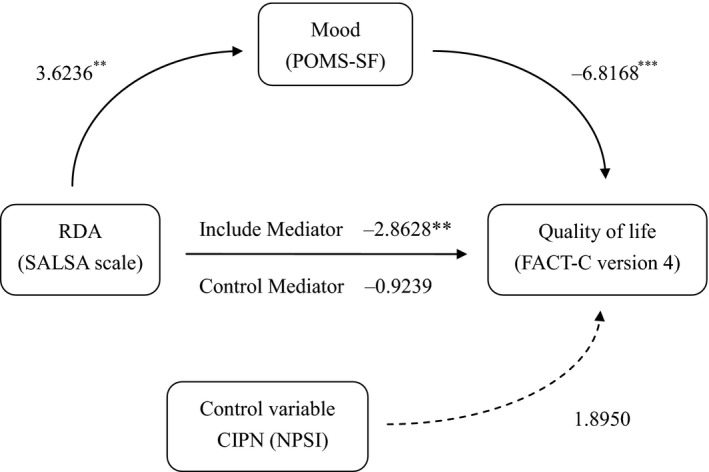
Mood mediates the effect of RDA on QoL. ^**^ *P* < 0.01; ^***^ *P* < 0.001. QoL, quality of life; RDA, restriction of daily activity

The mediation model of RDA and mood on QoL showed in Table 3 that RDA was positively related to mood (*t* = 3.6236, *P* < 0.01), and mood had a significant negative association with QoL (*t* = −6.8168, *P* < 0.001). Under the inclusion of mood, the total effects of RDA had a significantly negative relationship with QoL (*t* = −2.8628, *P* < 0.01). There was no significant relationship between RDA and QoL when the mood (mediator) was the control (*t* = −0.9239, *P* > 0.05). CIPN was the control covariate, with no significant relationship with QoL (*t* = 1.8950, *P* > 0.05). In conclusion, the 38.48% variance of the model can explain the total mediation of mood on the effect of RDA on QoL in controlling CIPN (Figure 1). The mediation model of mood and RDA on QoL also showed in Table 3. Because model I was included in 95% CI, the mediation model was excluded.

## 4 DISCUSSION

The aim of this study was to investigate the relationships among RDA, mood, and QoL in colorectal cancer patients with CIPN. In this study, the patients’ characteristics of age and gender were similar to the global statistical data.^1^ A previous study revealed that CIPN regardless of concurrent RDA may induce a poorer mood or QoL.^5^, ^8^ In this study, paroxysmal neuropathic pain and dysesthesia/paresthesia were associated with self‐care and dexterity of hand, a finding that was also reported previously.^8^, ^9^ For the first time, the present study revealed significant correlations of RDA with mood and QoL, compared with mild‐to‐moderate correlations of CIPN with mood and QoL. RDA likely had a stronger influence on mood and QoL than CIPN because only patients with mild‐to‐moderate CIPN were enrolled. Patients with severe CIPN should have been included in the study, but it was a challenging issue in clinical practice. Previous studies supported persistent severe CIPN may be irreversible, consequently inducing permanent RDA.^29^, ^30^ Thus, patients with severe CIPN were omitted from oxaliplatin‐based chemotherapy in clinical settings, and so would be excluded from the criteria of this study.

Additionally, most of the patients with mild‐to‐moderate CIPN had mild RDA. CIPN and RDA were associated with negative mood. Thus, spontaneous neuropathic pain, paroxysmal neuropathic pain, evoked neuropathic pain, and dysesthesia/paresthesia, as well as restrictions in foot mobility, self‐care, work of hand, and dexterity of hand lead to many types of emotional distress, such as tension, anxiety, depression, dejection, anger, hostility, vigor, activity, fatigue, inertia, confusion, and bewilderment. Similar findings revealed that RDA in patients with CIPN may show annoyance, irritability, weariness, anger, frustration, and depression.^10^ Negative mood was found to possibly induce poorer QoL in this study. Similar findings of relationships between negative mood and QoL have been reported previously.^15^, ^16^, ^17^

QoL is an endpoint of care for and treatment of cancer patients. According to the findings by hierarchical multiple linear regression analysis in this study, RDA, and negative mood can impact QoL more than CIPN. CIPN showed no significant effect on QoL. Thus, CIPN was set to be a covariate during mediation testing among RDA, mood, and QoL. Furthermore, negative mood can totally influence the effect of RDA on QoL. The two viewpoints are new findings in this study. Tracing the possible reason, the population in this study had a lower severity of CIPN and RDA, which may lead to fewer effects on QoL. Negative mood also commonly occurred in colorectal cancer patients. Although mood may overlap with the emotional well‐being of QoL, the overlaps were not conflicting. Under the operational definition of concept in instruments, POMS‐SF measured the negative mood by the frequency of the patients’ adjective concept, and FACT‐C version 4 evaluated the QoL by the frequency of events.^24^, ^25^, ^26^, ^27^ When measuring the operational definition of a concept, each sub‐concept should not be divided. Based on the same reason, NPSI evaluated the severity of pain by numerical measure and SALSA scale measured bivariate events, which were also different from the one of FACT‐C version 4.

By the mediation effect analysis, a new finding was noted in the study. Mood was a total mediator of RDA in QoL with the covariate CIPN. Thus, managing CIPN and RDA may not significantly improve QoL in colorectal cancer patients. Until their emotional problems are resolved, patients may continue to experience reduced QoL. This finding could explain why physicians were less concerned with the influence of CIPN in clinical practice.^10^, ^11^ It is also possible that there was no actual influence of mild‐to‐moderate CIPN or mild RDA on QoL. Based on our findings, seriously evaluating patients’ emotional status is necessary when caring for colorectal cancer patients with CIPN and RDA. Another one innovative viewpoint should be discussed, which is the possibility of a pathway from CIPN induced RDA, and then mood on QoL. In the fact, the pathway ever be analyzed by this study researchers, but its results excluded the routine. The cause was supposed to be a limitation in the population with mild‐to‐moderate CIPN. Another probability of limitation was methods of evaluating peripheral neuropathy. The methods of nerve conduction studies and quantitative sensory testing were not used in this study because there was no proper evaluation tool.^31^

Overall, the results of this study indicated that RDA in colorectal cancer patients with CIPN can induce a negative mood, which can then interfere with QoL. To avoid irreversible symptoms and maintain the treatment principle of do‐no‐harm, care specialists should conduct comprehensive evaluation and effective management of CIPN and RDA. The findings in this study strongly caution that care specialists must also actively evaluate and support patients’ emotional status, which may strongly promote QoL. Nevertheless, preventing irreversible CIPN, avoiding permanent RDA and maintaining mental hygiene are recommended to care specialists for the purpose of promoting QoL in colorectal cancer patients with CIPN. Finally, there were strong suggestions in clinical practice to evaluate patients’ mood status in daily routine and set up a criteria for consultation of social workers, psychologists, and psychiatrists.

## 5 LIMITATIONS

Participation was limited to colorectal patients with mild‐to‐moderate oxaliplatin‐induced peripheral neuropathy at two hospitals in Taiwan. The study had a cross‐sectional design that did not allow generalized findings throughout the longitudinal treatment course. In addition, multiple linear regression analysis could predict a trend between variables but could not prove the real cause effect between variables.

## 6 CONCLUSION

Based on the findings in this study, CIPN is a confounder of the relationships among RDA, mood, and QoL in colorectal cancer patients. Although the correlations between CIPN, mood, RDA, and QoL were around mild to moderate, all of them were significant. Mood is the strongest factor impacting QoL, especially in patients with mild‐to‐moderate CIPN and mild RDA. Managing and helping patients with CIPN and RDA as well as adjust their negative mood are very important in promoting QoL in colorectal cancer patients receiving oxaliplatin‐based chemotherapy.

## CONFLICTS OF INTEREST

The corresponding authors are employed by educational institutes, and the first author and coauthors are employed by the hospital. They are educators and primary care specialists with no conflicts of interest.
